# Supplementary material for: Enhanced Cytotoxic Effect of Doxorubicin Conjugated to Glutathione-Stabilized Gold Nanoparticles in Canine Osteosarcoma—In Vitro Studies
Source: Molecules. 2021 Jun 8;26(12):3487. doi: 10.3390/molecules26123487 (PMC8227216; doi:10.3390/molecules26123487)

SUPPLEMENTARY MATERIALS

Supplementary materials S3. Contrast-phase images of osteosarcoma cell lines: D17, x4, scale bar 200 µm (A), D17, x20, scale bar 50 µm (B), U2OS, x4, scale bar 200 µm (C), U2OS, x20, scale bar 50 µm (D), treated with increasing doses of Au-GSH, Au-GSH-Dox and Dox. Signs of apoptosis were observed: detached cells (red, long arrows), cell shrinkage (red arrowheads), membrane blebbing (red asterisks).

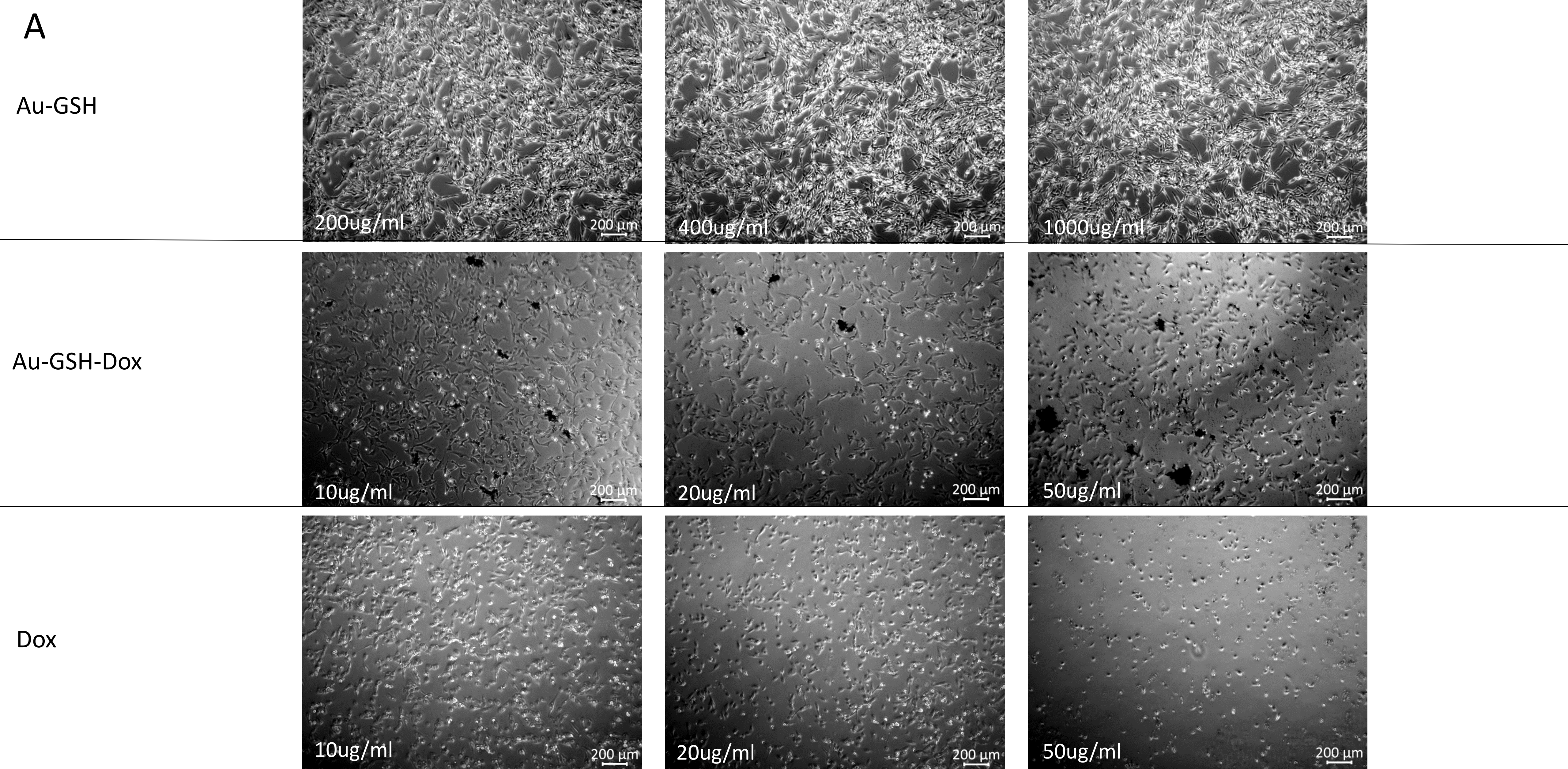

SUPPLEMENTARY MATERIALS

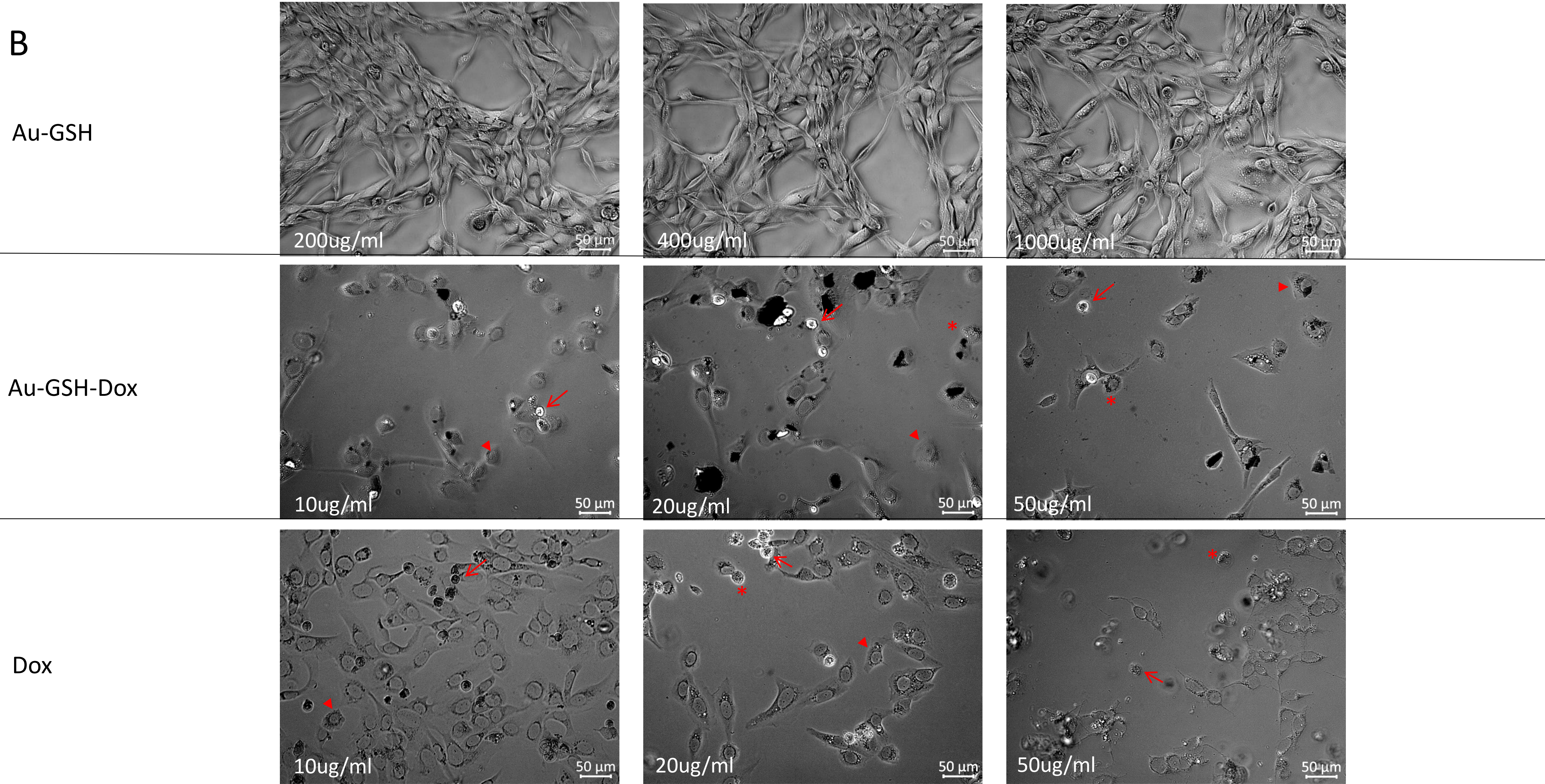

C

Au-GSH

Au-GSH-Dox

Dox

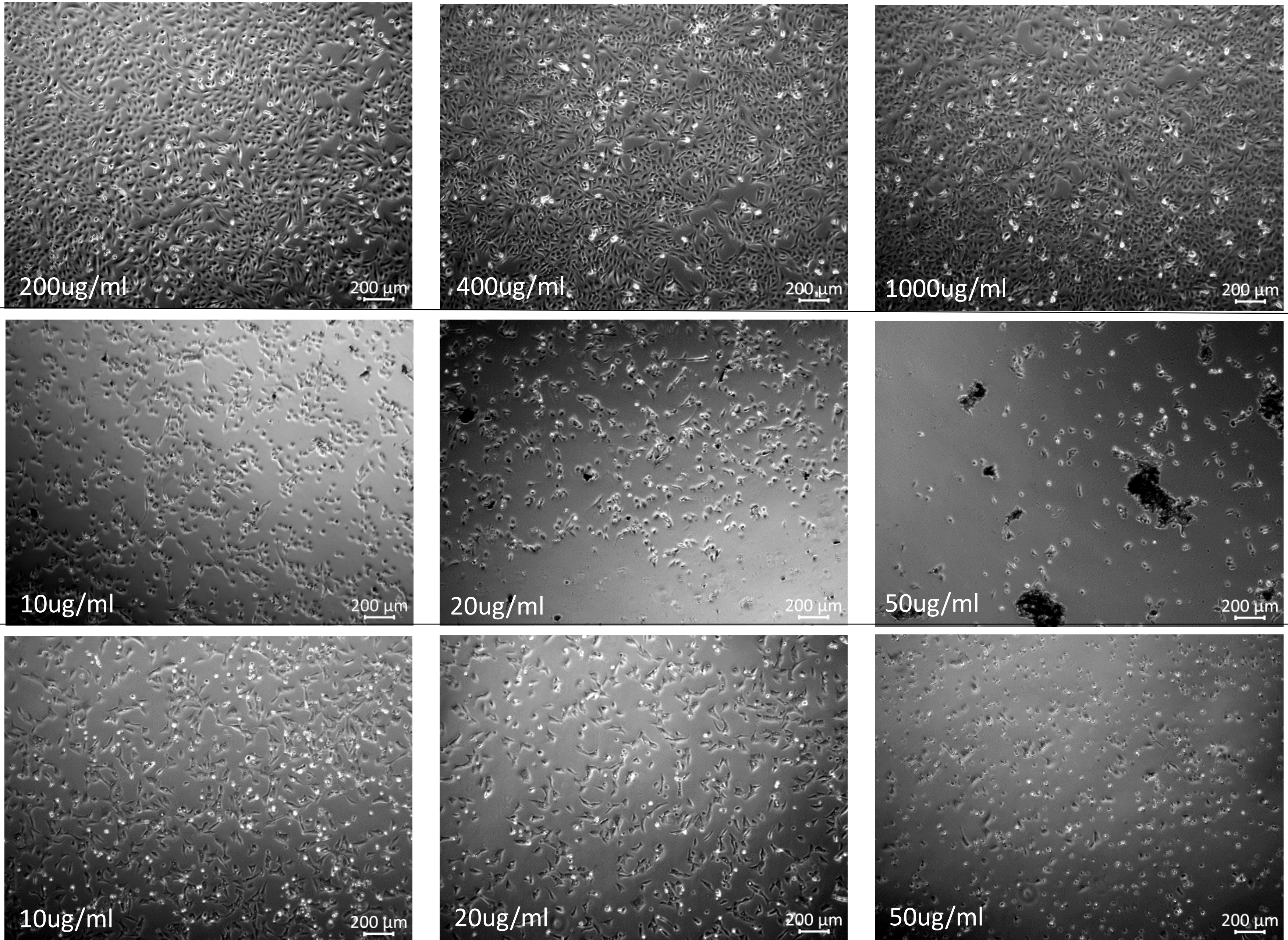

SUPPLEMENTARY MATERIALS

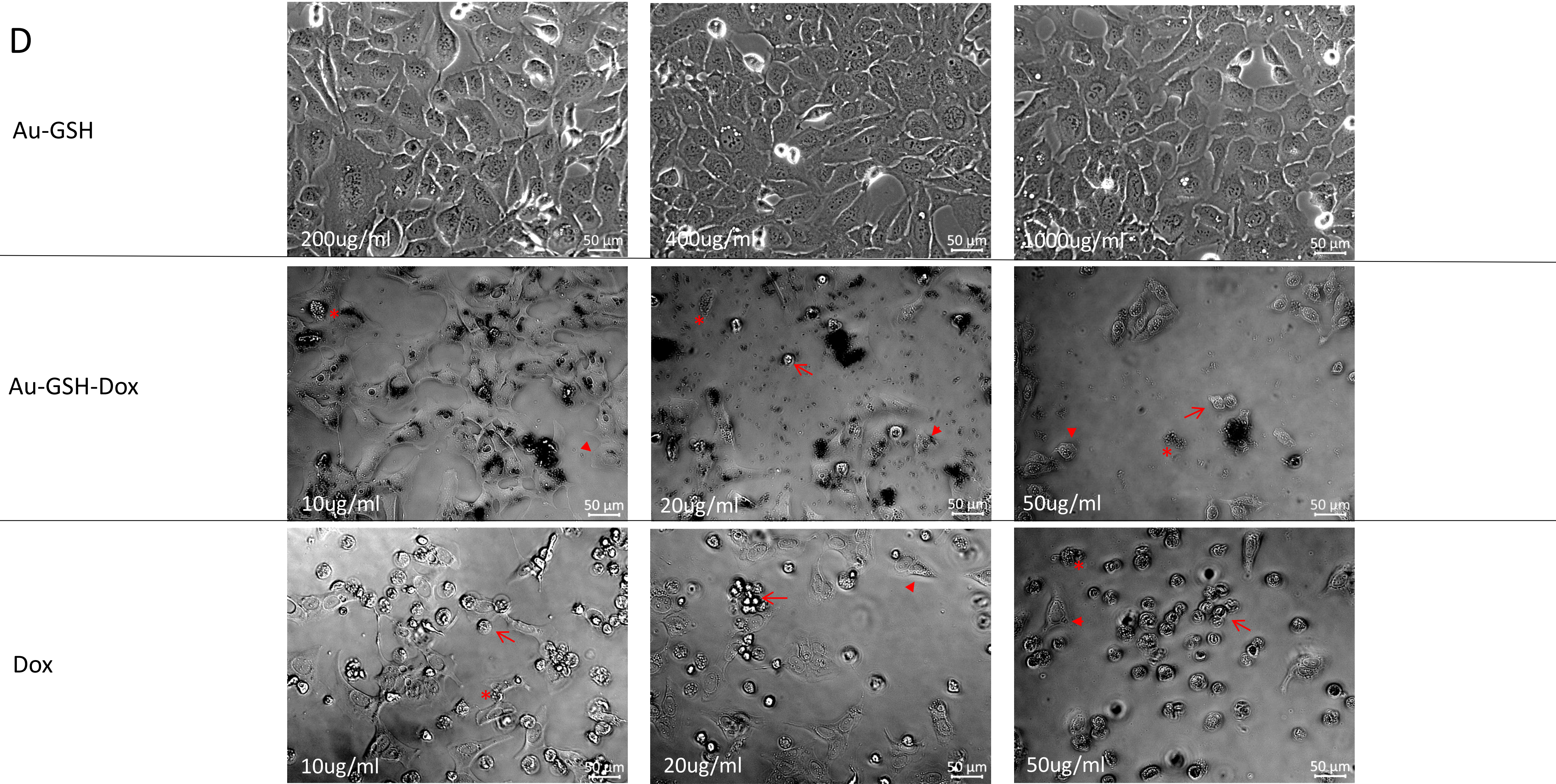

Supplement: Supplementary file 1 [file molecules-26-03487-s001.zip › Supplementary materials 3 proof.pdf]
