# Supplementary material for: Enhanced Cytotoxic Effect of Doxorubicin Conjugated to Glutathione-Stabilized Gold Nanoparticles in Canine Osteosarcoma—In Vitro Studies
Source: Molecules. 2021 Jun 8;26(12):3487. doi: 10.3390/molecules26123487 (PMC8227216; doi:10.3390/molecules26123487)

SUPPLEMENTARY MATERIALS

Supplementary materials S1. MTT assay - cell viability (% +/- SEM) after the 24h treatment with Au-GSH for D17 (A) and U2OS (B) cell lines.

A

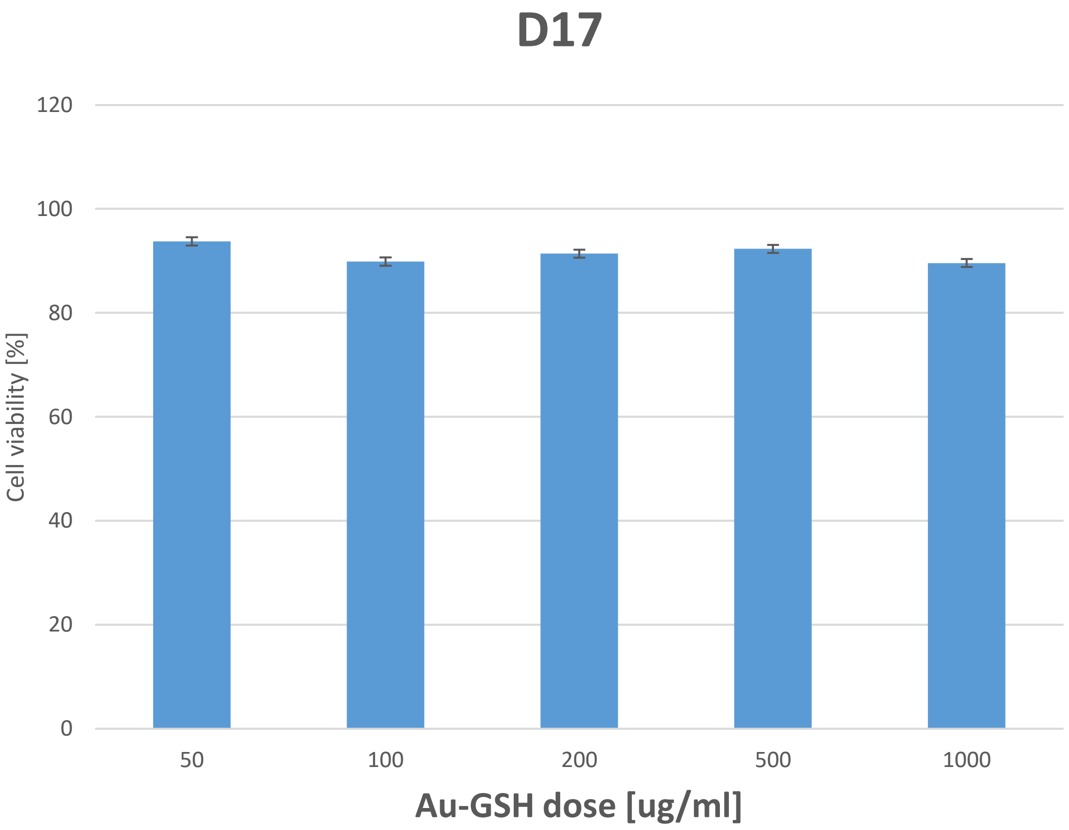

B

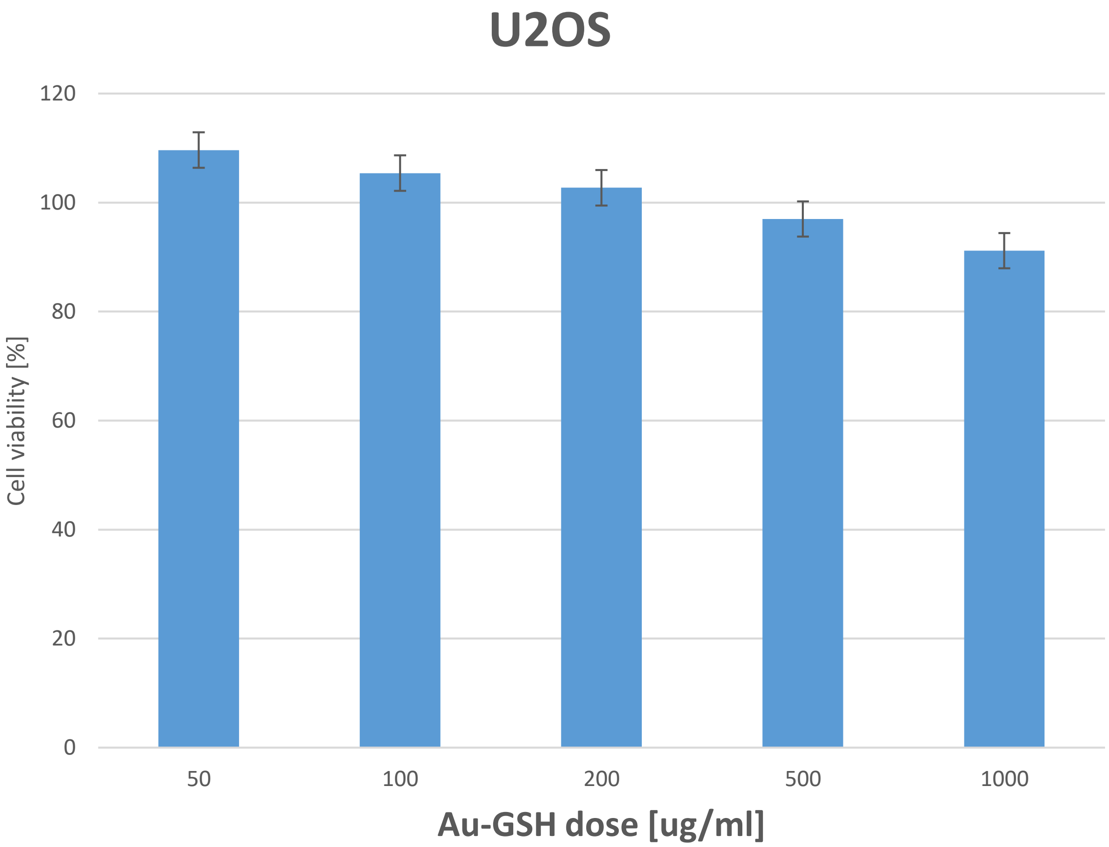

Supplement: Supplementary file 1 [file molecules-26-03487-s001.zip › Supplementary materials 1 proof.pdf]
