# Supplementary material for: Enhanced Cytotoxic Effect of Doxorubicin Conjugated to Glutathione-Stabilized Gold Nanoparticles in Canine Osteosarcoma—In Vitro Studies
Source: Molecules. 2021 Jun 8;26(12):3487. doi: 10.3390/molecules26123487 (PMC8227216; doi:10.3390/molecules26123487)

SUPPLEMENTARY MATERIALS

Supplementary materials S2. Effect of Au-GSH on the mortality (% +/- SEM) of tested cell lines: D17 (A) U2OS (B).

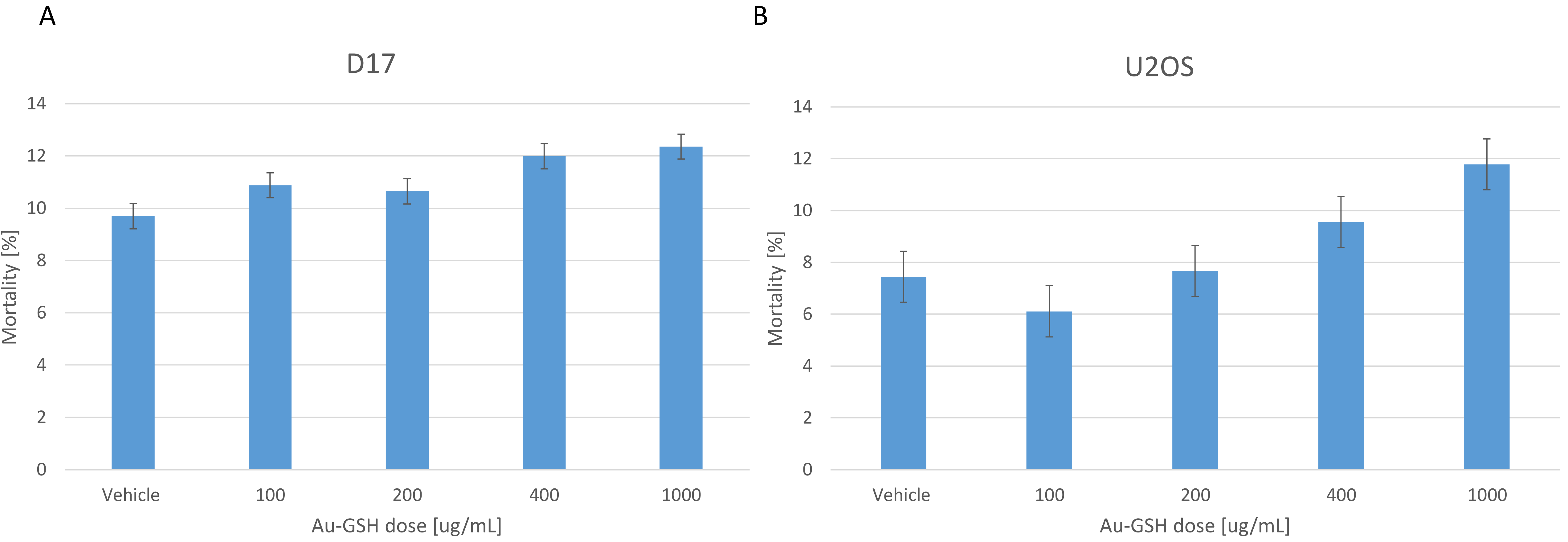

Supplement: Supplementary file 1 [file molecules-26-03487-s001.zip › Supplementary materials 2 proof.pdf]
